# From Preparticipation Screening to Diagnosis: Long-Term Outcomes of Athletes with Ventricular Repolarization Abnormalities and Normal Echocardiography

**DOI:** 10.3390/jpm16030136

**Published:** 2026-03-01

**Authors:** Massimiliano Bianco, Fabrizio Sollazzo, Stefania Manes, Andrea Giovanni Cristaudo, Gloria Modica, Riccardo Monti, Michela Cammarano, Paolo Zeppilli, Vincenzo Palmieri

**Affiliations:** Sport Medicine Unit, Fondazione Policlinico Universitario Agostino Gemelli IRCCS, 00168 Rome, Italy; massimiliano.bianco@policlinicogemelli.it (M.B.); stefania.manes01@icatt.it (S.M.); andrea.cristaudo01@icatt.it (A.G.C.); gloria.modica@guest.policlinicogemelli.it (G.M.); riccardo.monti@policlinicogemelli.it (R.M.); michela.cammarano01@gmail.com (M.C.); paolo.zeppilli@unicatt.it (P.Z.); vincenzo.palmieri@unicatt.it (V.P.)

**Keywords:** athletes, athlete’s heart, preparticipation screening, ventricular repolarization abnormalities, T waves inversion, electrocardiography, stress test, echocardiography, follow-up

## Abstract

**Background/Objectives**: Ventricular repolarization abnormalities (VRA) represent a grey area in athlete screening: some patterns are physiological, while others are precursors to heart disease. Objective: to clarify the natural history of VRA and the associated factors of structural diagnosis. **Methods**: Retrospective observational single-center study of athletes with resting or stress VRA at the first evaluation, with normal echocardiography; minimum follow-up of 2 years. Clinical data, resting and stress ECG, echocardiography, and selective advanced imaging throughout follow-up were collected. Primary outcome: cardiovascular diagnosis at follow-up; time-to-event analysis and associations between ECG characteristics and diagnosis. **Results**: Fifty-three athletes (mean age 22.2 ± 9.2 years; 92.5% male) were included; 60.4% had resting VRA, and 100% had exercise-induced VRA at baseline. Over 7.3 ± 4.5 years, 28/53 (52.8%) received a diagnosis; median time-to-detection was 7.0 years (95% CI 6.0–not reached); RMST10 was 6.7 years (95% CI 5.7–7.7). Diagnoses included hypertrophic cardiomyopathy (24.5%), non-ischaemic left-ventricular scar (11.3%), myocardial bridging (7.5%), hypertensive remodelling (5.7%), coronary anomaly (1.9%), and ventricular pre-excitation (1.9%). Persistence of resting VRA from baseline to follow-up was more frequent in athletes with a final diagnosis (*p* = 0.01), whereas topography and exercise-induced abnormalities did not discriminate groups. Advanced imaging contributed substantially to case ascertainment. No major adverse cardiovascular events have been identified throughout follow-up. **Conclusions**: In athletes with screening-detected VRA and normal echocardiography, persistence of resting VRA was associated with higher detection of a cardiovascular diagnosis, while exercise-induced changes alone show limited diagnostic yield. The long median time-to-detection supports prolonged, pre-planned surveillance, with priority for advanced imaging in profiles with persistent abnormalities. These findings align with a risk-adapted, personalized management strategy in sports cardiology.

## 1. Introduction

Regular, high-intensity physical training induces a series of morphological and functional adaptations in the cardiovascular system known as “athlete’s heart”, an expression of physiological and reversible remodelling that optimises the haemodynamic response to exercise [1,2]. In recent decades, the definition of athlete’s heart has evolved from a predominantly morphological concept to an integrated model that includes anatomical, physiological, metabolic, and (in part) genetic parameters, with inter-individual variability determined by age, sex, ethnicity, and type/amount of workload [3]. However, the distinction between physiological adaptation and underlying heart disease is not always easy: there is a large “grey area” where ECG and imaging findings may overlap with those seen in cardiomyopathies, with significant implications for athlete safety and competitive sport eligibility [4,5].

In any case, the 12-lead electrocardiogram still represents the backbone of pre-participation assessment for athletes, an examination that has significantly reduced the incidence of sudden death in athletes over the years [6,7].

Despite these advances, the most challenging interpretative problem remains that of ventricular repolarisation abnormalities (VRA), represented by ST-segment modifications and, above all, T-wave inversion (TWI). VR in athletes is a “grey area”: it may reflect functional modulations related to autonomic tone and training-induced electrical remodelling, but it may also be an early warning sign of structural heart disease (hypertrophic, arrhythmogenic, non-compaction) or channelopathies. The evolution of interpretative criteria—from the Seattle and Refined Criteria to the 2017 International Criteria—has progressively reduced false positives, emphasising the importance of context (age, sex, ethnicity), topography (location/extension of TWI), and integration with second-level tests [8]. In particular, TWI confined to the right precordial leads in young people and individuals of African/Afro-Caribbean origin may be physiological, while inversions in the lateral or inferolateral leads are more frequently associated with heart disease and require a structured work-up (echocardiography and often cardiac magnetic resonance imaging, CMR) [9,10,11,12].

Resting ST-segment depression is uncommon in athletes and should raise suspicion of structural heart disease in the absence of confounders such as bundle-branch block, ventricular pre-excitation, electrolyte disorders, stroke, or drug effects [13]. Although these elements improve the specificity of screening, they do not eliminate the need for longitudinal surveillance in non-decisive cases.

A further source of complexity is provided by the stress ECG, an essential component of competitive sports medical examinations in Italy [14] and often performed as a second-level assessment in athletes with arrhythmias or suspected heart disease [15]. Exercise modulates frequency, autonomic tone and haemodynamic load, which can bring out or transform the morphology of repolarisation: ST-segment modifications are typically related to coronary artery disease, a widespread issue, not only in the general population [16], but also among older athletes (i.e., “master” athletes) in the sports field, whereas the occurrence of TWI during stress ECG may be associated not only with inducible ischaemia (typical of coronary artery disease, but also of prominent conditions in sports cardiology, such as anomalous origin of coronary arteries and myocardial bridging) but also with heart diseases of very different aetiology, such as hypertrophic and dilated cardiomyopathies, myopericarditis (acute or in sequelae), mitral valve prolapse and arrhythmogenic cardiomyopathy [17,18]. These abnormalities and their changes induced by stress test, however low in specificity and sensitivity, represent a ‘red flag’ for careful observation in the context of pre-participation screening and lead to further in-depth examinations (in relation to the overall clinical picture, e.g., symptoms, arrhythmias) and the inclusion of the athlete in close follow-up [19].

Despite shared guidelines and criteria, areas of clinical uncertainty remain. The most significant concerns the natural history of athletes who present with VRA in the absence of diagnosis and with an echocardiogram initially within normal limits [20]: (i) among athletes with screening-detected VRA and normal echocardiography, what is the incidence and time-to-detection of cardiovascular diagnoses? (ii) Do resting vs. stress-induced VRA at baseline, and their longitudinal behaviour (persistence/attenuation/new onset), differentiate those who will receive a diagnosis? (iii) How do ECG features contribute to risk stratification within this longitudinal framework? The answers to these questions are essential for calibrating follow-up and further investigation in a proportionate manner, avoiding both overdiagnosis and underestimation of risk.

In light of these factors, this study aims to investigate a population with VRA at rest and/or during exercise that showed no signs of structural heart disease at initial assessment, with the aim of describing the natural history of VRA patterns and their relationship with the identification of cardiovascular disease diagnoses. The ultimate goal is to propose clearer clinical criteria and follow-up pathways that are truly proportionate to risk, improving the appropriateness of screening and health protection in sport.

## 2. Materials and Methods

This involved a retrospective, single-centre observational study conducted at our Sports Medicine Unit. All consecutive subjects evaluated for pre-participation screening from 2006 to 2022 were carefully examined, and those with rest and/or stress ventricular repolarisation abnormalities (VRA) at the first evaluation were included, provided they also had a clinical-instrumental follow-up of ≥24 months.

This retrospective ancillary analysis derives from an institutional pathway for athletes referred for VRA prompting evaluation for suspected hypertrophic cardiomyopathy, conducted after Ethics Committee approval (protocol ID 1920; 15 February 2018). Following diagnostic work-up, athletes received a specific diagnosis or remained phenotype-negative (‘no diagnosis’), the latter retained as an internal comparator. Data collection took place from 2006 to 2018.2, and follow-up data were collected from 2018.3 to 2022. All data processing and analysis were performed after ethical approval was obtained. The following inclusion criteria were applied: (I) non-master athletes (<40 years old for men, <50 years old for women); (II) documented participation in competitive or non-competitive sports; (III) no evident diagnosis of structural or arrhythmic heart disease at initial assessment (including, therefore, an echocardiographic assessment within normal limits); (IV) absence of evident non-cardiovascular pathologies affecting ventricular repolarisation on ECG (e.g., alterations in blood electrolytes, uncompensated thyroid disorders, subarachnoid haemorrhage, pulmonary embolism, severe anaemia); (V) no treatment with cardioactive drugs and documented or suspected use of anabolic/antiandrogenic steroids in the last 12 months. All subjects with incomplete clinical or instrumental data at the initial assessment were excluded.

The sports practised were classified according to the taxonomy of the European Society of Cardiology, distinguishing between sports of skill, power, endurance, and mixed sports [4].

TWI was defined as the presence of negative T waves ≥ 1 mm in depth in two or more contiguous leads (excluding leads aVR, III and V1) in an anterior, lateral, inferolateral or inferior territory (except leads V1–V4 in black athletes and leads V1–V3 in all athletes aged < 16 years) as per international criteria (8). The topography of TWI has been classified as: anterior (right precordial/mid-anterior), lateral (V4–V6, DI, aVL), inferior (DII, DIII, aVF), and combinations of these.

Similarly, ST-segment depression was defined as the presence of an ST-segment below the isoelectric PR segment greater than 0.05 mV (0.5 mm) in two or more leads (8).

Exercise-induced VRA have been defined as the appearance or significant accentuation of ST-T alterations during exercise testing and/or recovery compared to baseline (that is, ≥1 mm additional T-wave negativity in ≥2 contiguous leads and/or extension to ≥1 additional contiguous lead territory or new or increased horizontal/downsloping depression ≥ 0.5 mm in ≥2 leads relative to baseline). The abnormality criteria were drawn from the guidelines on the subject [21]. Other additional electrocardiographic parameters considered informative for patient assessment were noted (e.g., PR interval, Q waves).

At follow-up, VRA at rest and/or during exercise were indicated in cases where previously detected abnormalities persisted and where a newly detected abnormality appeared. Follow-up rest and stress ECGs were performed at variable, clinically driven intervals according to symptoms, training load, and interim findings.

Of note, persistence of resting VRA was defined a priori as the persistence of the same pattern across ≥ 2 follow-up visits or ≥12 months, with qualitatively similar morphology (territory) and no net attenuation below diagnostic thresholds.

The reports were carried out by three operators with experience in sports cardiology; any discrepancies in interpretation were resolved by consensus in order to ensure consistency and robustness in the assessment.

All participants underwent maximal exercise testing on a cycle ergometer or treadmill, or alternatively cardiopulmonary exercise testing on the same type of ergometer. The protocols were selected from those validated according to the international literature and the experience of our Centre and individualised based on age, training level, and clinical profile. During the examination, the appearance or modification of ventricular repolarisation abnormalities, the possible onset of supraventricular or ventricular arrhythmias, evidence of ischaemia, and the appearance of further electrocardiographic abnormalities were monitored. These data were integrated with the baseline ECG findings to outline the dynamics of ventricular repolarisation.

Cardiac imaging included, in all subjects, echocardiography performed according to current standards, with particular attention to the measurement of ventricular thicknesses and diameters, assessment of left and right ventricular geometry and systolic and diastolic function, any valve defects, and alterations in the pericardium.

Additional cardiological tests such as 24 h Holter ECG monitoring, ambulatory blood pressure monitoring, blood chemistry tests, computerized coronary tomography angiography (CCTA), and cardiac magnetic resonance (CMR) were performed based on clinical judgement in each individual case. Where applicable, additional third-level examinations were also taken into consideration, such as electrophysiological studies with electro-anatomical mapping, endomyocardial biopsy, genetic analysis, and coronary angiography, where required by the clinical picture or aimed at resolving aetiological doubts; the relevant results were incorporated into the decision-making process to confirm or rule out specific disease hypotheses.

At each follow-up, the possibility of identifying a diagnosis was assessed by integrating all the clinical and instrumental data collected for each patient with the main cardiological guidelines available. The presence or absence of a diagnosis at follow-up was identified qualitatively and temporally and was also graded on the basis of four ordinal categories (none, possible, probable, certain) according to the level of diagnostic confidence achieved.

### Statistical Analysis

Continuous variables were described as median [IQR] or mean ± SD, depending on the distribution; categorical variables were described as counts and percentages.

The primary outcome was the occurrence of cardiovascular diagnosis during follow-up (yes/no) and the relative time-to-detection. In time-to-event analyses, those who did not reach the outcome threshold were censored at the last clinical contact.

The cumulative incidence of diagnosis was estimated as proportion x/N with 95% CI (Wilson method).

To assess the association between the main electrocardiographic characteristics (in particular, type and topography of VRA) and the presence of diagnosis in follow-up, 2 × 2 tables were constructed, and crude odds ratios (ORs) with 95% CI were estimated. For expected cells < 5, Fisher’s exact test was used.

To verify whether the prevalence of TWI changed between baseline (T0) and last follow-up (FU), the McNemar test for paired data was used.

Time-to-detection was analyzed using Kaplan–Meier curves of the probability of remaining diagnosis-free (95% CIs). Administrative censoring was pre-specified at τ = 10 years to ensure estimator stability (adequate numbers at risk) and interpretability.

Given the limited number of events, we refrained from using over-parameterized models to avoid overfitting.

The analyses were conducted with all two-tailed tests and a significance threshold of *p* < 0.05. The analyses were performed with JASP (version 0.9.5).

## 3. Results

During the period of interest, 4695 non-master athletes underwent pre-participation screening for competitive sports, including resting and exercise ECG and echocardiography. Among these, 167 athletes had resting or stress VRA, but only 53 athletes (49 males, 92.5%) met the inclusion criteria. Mean age at baseline assessment was 22.2 ± 9.2 years, and most of the athletes practiced mixed sports (40, 75.5%), followed by endurance sport (11, 20.8%), power sport (4, 7,5%), and skill sports (1, 1.9%). Rest, stress ECG, and echocardiographic features of the sample at baseline assessment are presented in Table 1.

Specifically, at the baseline resting electrocardiographic assessment, 32 subjects (60.4%) had VRA. The topographic distribution of VRA showed a predominantly lateral pattern (25 out of 32, 78.1%), followed by anterior (9/32, 28.1%) and inferior (11/32, 34.3%), with numerous mixed forms, particularly inferior-lateral pattern (10/32, 31.3%). At follow-up, VRA persisted in 29 athletes (54.7%), while it was absent in 24 (45.3%). The most frequently involved leads remained the lateral (24/29, 82.8%) and inferior-lateral (9/29, 31.0%) leads, while isolated inversions in the anterior or inferior leads were less common. With regard to the stress ECG, all 53 subjects (100%) presented VRA at the initial assessment, with a clear predominance of inferior-lateral patterns (26, 49.1%). At follow-up, VRA remained present in 50 of cases (94.3%), with a persistent predominance of inferior-lateral patterns (33, 62.3%).

**Figure 1:**
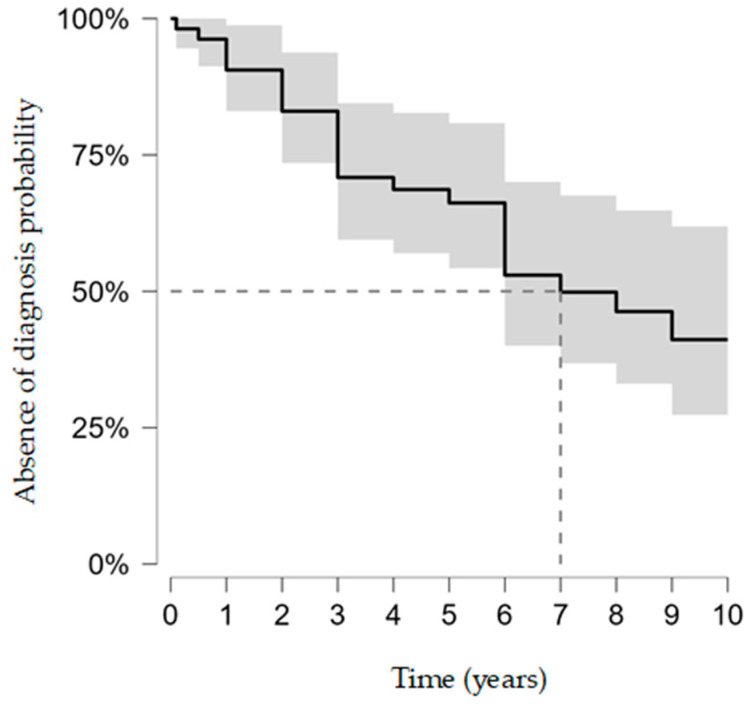
Kaplan–Meier time-to-detection of cardiovascular diagnosis (probability of remaining diagnosis-free; 95% CIs). Event = first confirmed diagnosis; censoring at last follow-up. Administrative censoring was pre-specified at 10 years for estimator stability and interpretability.

Mean follow-up was 7.3 ± 4.5 years. Overall, 28 athletes (52.8%) received a diagnosis during follow-up, while 25 (47.2%) remained diagnosis-free at last contact. Among those diagnosed, the diagnostic grade was classified as certain in 35.8% of cases (n = 19), probable in 9.4% (n = 5), and possible in 7.5% (n = 4). Kaplan–Meier time-to-detection analysis yielded a median of 7.0 years (95% CI 6.0–not reached). The restricted mean time without diagnosis at 10 years (RMST10) was 6.7 years (95% CI 5.7–7.7) (Figure 1).

**Figure 2:**
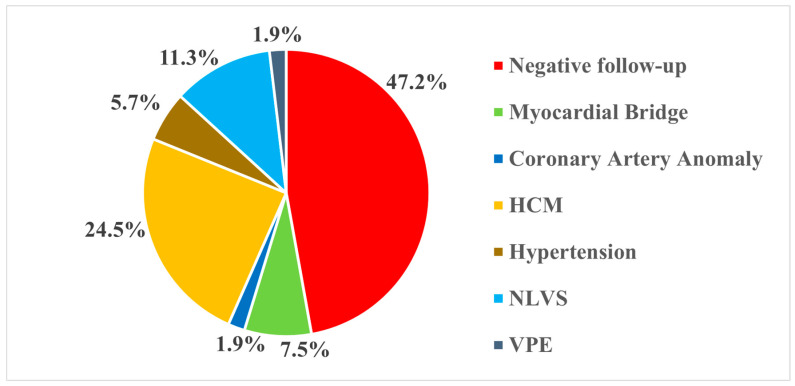
Distribution of diagnoses at follow-up. HCM: Hypertrophic Cardiomyopathy; NLVS: Nonischemic Left Ventricular Scar; VPE: Ventricular Pre-Excitation.

Among the diagnoses achieved, Hypertrophic Cardiomyopathy (HCM) (13, 24.5%) and Non-Ischemic Left Ventricular Scar (NLVS) (6, 11.3%) accounted for more than one-third of cases (35.8%), followed by myocardial bridge (4, 7.5%), arterial hypertension with ventricular remodeling (3, 5.7%), coronary artery anomaly (1, 1.9%), and ventricular pre-excitation (1, 1.9%) (Figure 2).

**Figure 3:**
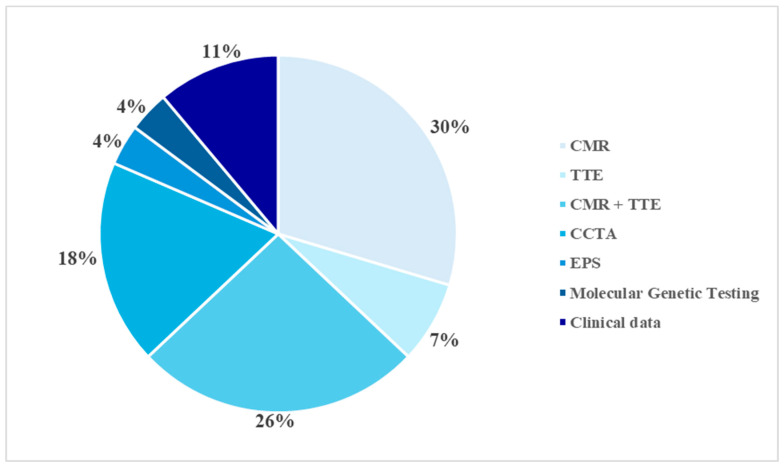
Diagnostic methods. CMR: Cardiac Magnetic Resonance; TTE: Transthoracic Echocardiography; CCTA: Computed Coronary Tomography Angiography; EPS: Electrophysiological Study.

In the sample examined, the diagnosis was ascertained using different diagnostic methods, employed individually or in combination, depending on the complexity of the clinical picture and initial findings. CMR imaging was the main diagnostic tool, proving decisive in identifying the pathology in eight cases (28.6%). In seven patients (25%), the diagnosis was made through a combined echocardiographic and CMR evaluation. CCTA was the decisive method in five cases (17.9%), while in two subjects, echocardiography was sufficient to confirm the diagnosis (7.1%). In a further three cases (10.7%), the diagnosis was reached through an integrated assessment of echocardiographic and clinical data. Finally, in one case each (3.6%), the diagnosis was obtained through electrophysiological study and molecular genetic testing (Figure 3).

In terms of symptoms, the majority of subjects were asymptomatic at the first assessment (52.8%, n = 28), while 25 reported a variable combination of cardiovascular symptoms (mainly palpitations and angina). At the follow-up assessment, the proportion of asymptomatic subjects increased to 67.9% (n = 36). Only one-third of subjects maintained or developed persistent or recurrent symptoms, with no significant differences between the group with and without a final diagnosis (*p* > 0.05).

Analysis of the resting and exercise VRA profiles at the two timepoints between individuals with and without a diagnosis of heart disease identified a statistically significant difference in the persistence of VRA at rest between baseline and follow-up evaluation (*p* = 0.01), whereas other ECG features do not show any relevant difference. Even the analysis of arrhythmic activity, assessed during stress ECG, did not reveal any significant differences between subjects with and without a final diagnosis (*p* > 0.05, Table 2).

It is important to note that no major cardiovascular adverse events were identified in the entire population considered for the whole follow-up period.

## 4. Discussion

The study of resting and stress ECGs is still a cornerstone of pre-participation screening of athletes, as these are useful, relatively inexpensive, and readily available tools for the early detection of suspected heart conditions that may be associated with a risk of adverse sports-related cardiovascular events. However, the distinction between physiological VRA and those representing early manifestations of cardiovascular disease remains one of the most complex and debated areas in contemporary sports cardiology [19].

In our study, which looked at a group of athletes with VRA found during screening and with normal baseline echocardiograms, persistence of resting VRA over time was associated with subsequent case ascertainment. The presence of VRA at rest that does not resolve at follow-up is consistently associated with subsequent diagnosis of heart disease; conversely, resolution or attenuation of VRA during follow-up is associated with a lower probability of diagnosis. This result reaffirms once again the importance of longitudinal screening over an adequate period of time: the dynamics of VRA between visits is a determining factor in the identification of heart disease, compared to an isolated finding, particularly where, as in our case, the morphological assessment initially gave a negative result.

The second important aspect that emerges from this study is how the finding of significant VRA corresponded to a higher diagnostic yield in this selected cohort: more than half of the individuals included in our case series were diagnosed with heart disease. This element reveals some new aspects compared to similar case series previously published [11,20], once again pointing, on the one hand, to the need for maximum attention to these abnormal electrocardiographic findings and, on the other, to the need for sometimes very prolonged follow-up before the actual pathological conditions can be identified. The median time-to-detection of around seven years and the lack of definition of the upper limit of the confidence interval indicate that a significant proportion of cases emerge beyond the commonly adopted surveillance intervals. These elements, therefore, do not allow us to rule out the late emergence of a diagnosis of heart disease in the presence of significant VRA, even in the presence of initial echocardiographic findings within the normal range, suggesting the need for prolonged follow-up over time and sensitivity to the appearance of further abnormal findings (complex ventricular arrhythmias, symptoms, new echocardiographic anomalies).

Exercise-induced VRA at first evaluation were omnipresent, but the proportion of subsequent diagnoses in the medium to long term is modest. Neither arrhythmic behaviour during exercise nor the mere positivity of the exercise test for VRA showed an association with further diagnosis; therefore, their presence per se did not stratify risk nor shorten time-to-detection of a cardiovascular diagnosis. In practical terms, an isolated positive exercise ECG for VRA without concomitant red flags did not add diagnostic discrimination and should not, by itself, prompt escalation to advanced imaging. In our sample, the ubiquity of exercise-induced VRA and clinically selective work-ups does not allow us to estimate specificity or false positive rates; however, our findings are consistent with external data reporting significant false positives in ExECG in athletes/low-risk individuals and in non-ischaemic conditions (e.g., myocardial bridging) capable of inducing ST-T alterations during exercise [22]. This observation supports data showing the low specificity of ST-T alterations in trained individuals and the hypothesis that these abnormalities often are a benign adaptive phenomenon linked to increased adrenergic tone and changes in ventricular load during exercise [23,24].

The anatomical distribution of VRA has been proposed as a potential discriminator between physiological and pathological patterns [25], with infero-lateral involvement generally considered more concerning than isolated anterior changes [26], particularly in young athletes [27]. In our series, topography (anterior vs. infero-lateral) did not show a statistically significant association with subsequent diagnosis. This null result should be interpreted with caution due to the limited number of our series, which does not allow us to analyse how topography interacts with other features—presence at rest, persistence over time, extent (number of involved leads), recovery behaviour, and ventricular arrhythmias. Clinically, our data suggest that topography alone should not drive escalation in the absence of persistent resting VRA or additional abnormal findings.

Obviously, what greatly influenced the diagnosis was the use of advanced investigative techniques (e.g., CMR, CCTA, genetic analysis), which represent the gold standard for diagnosis in their respective specific applications. However, the selection of candidates and the timing of these investigations are critical [12,28]: our data suggest that persistence of resting VRA should be a primary criterion to identify athletes who are most likely to benefit from targeted advanced imaging, considered within the overall clinical context. For these athletes, inclusion in an individualized, pre-specified surveillance programme appears essential and should be systematically implemented in sports cardiology services.

Overall, the data support a gradual and personalised approach to assessing athletes’ VRA, with a more stringent approach to follow-up and advanced investigations in cases of persistent VRA over time, whereas in cases of attenuation or resolution of findings, less intensive follow-up seems reasonable. This evidence is consistent with the principles of personalised medicine applied to athletes: the estimation of the risk of heart disease is derived from the integration of baseline electrocardiographic and echocardiographic variables, their behaviour over time, and the overall clinical context [29,30,31].

This study has some limitations that should be considered when interpreting the results. The sample is small and selected, resulting in limited power and poor generalizability to unselected populations. Given the observational design and clinically driven, non-uniform work-up (including selective access to level-III investigations), this study is subject to verification bias, whereby patients undergoing more intensive evaluation are more likely to receive a diagnosis; accordingly, time-to-detection estimates reflect the timing of scheduled investigations. For the same reason, the observed diagnostic yield likely overestimates that of unselected athletic populations, so absolute proportions should not be generalized beyond similar referral settings; this aspect must also be emphasised in view of the small sample size and the single-centre nature of this study. Subclinical disease at index cannot be reliably excluded because advanced imaging (CMR and CCTA) was not performed systematically at baseline; selective use based on suspicion may have introduced verification bias. ECG variables (location/extent of T wave inversion) may be subject to misclassification and interobserver variability, despite the precautions taken. Control of confounders is necessarily parsimonious due to the limited number of events, with possible residual confounding. Specifically, changes in training load, sport modality, and detraining were not systematically captured during follow-up; these factors can modulate ST–T morphology and may confound longitudinal ECG trajectories. The results of this study will therefore need to be confirmed in larger cohorts with standardized initial diagnostic and imaging protocols.

## 5. Conclusions

In a cohort of athletes with ventricular repolarization abnormalities (VRA) at screening and normal baseline echocardiograms, clinical outcome was associated with the persistence of VRA over time, rather than with the initial finding or exercise behavior alone. The long median time-to-detection (about 7 years) indicates that a significant proportion of cases emerge beyond commonly adopted surveillance horizons, reinforcing the need for prolonged, personalized, and pre-planned follow-up. In a setting where exercise-induced VRA are ubiquitous, their isolated presence is not clinically discriminative; escalation should be driven by persistent resting VRA and converging clinical/ECG findings rather than exercise positivity alone. Operationally, the data support a gradual and personalized approach: initial phenotypic characterization, longitudinal monitoring, and priority for level III investigations in profiles with persistent VRA and/or additional abnormal findings, with less intensive follow-up when attenuation or resolution is observed. These findings are consistent with the principles of precision medicine applied to athletes and outline a practical pathway that optimizes diagnostic performance while limiting unnecessary tests.

## Figures and Tables

**Table 1:** Electrocardiographic and echocardiographic results at first evaluation.

Parameters	Characteristics	N (% of the Sample)	Mean (SD)
**Electrocardiography**			
**Resting VRA**		**32 (60.4)**	
Resting VRA—topography			
	Anterior	9 (17.0)	
	Lateral	25 (47.2)	
	Inferior	11 (20.8)	
**Stress VRA**		**53 (100)**	
Stress VRA—topography			
	Anterior	19 (35.8)	
	Lateral	47 (88.7)	
	Inferior	40 (75.5)	
**Other ECG anomalies**		**28 (52.8)**	
Common		23 (43.4)	
Minor Uncommon		2 (3.8)	
	RBBB	1 (1.9)	
	Low voltages	1 (1.9)	
Major Uncommon		2 (3.8)	
	Pathological Q waves	2 (3.8)	
**Arrhythmias**		**11 (20.8)**	
SVA		5 (9.4)	
	Rare/Sporadic/Frequent	4 (7.5)/0/1 (1.9)	
VA		6 (11.3)	
	Rare/Sporadic/Frequent	3 (5.7)/2 (3.8)/1 (1.9)	
	Isolated/Complex	0/0	
	Monomorphic/Polymorphic	6 (11.3)/0	
	Common/Uncommon	3 (5.7)/3 (5.7)	
	Rest/Stress	3 (5.7)/2 (3.8)	
**Echocardiography**			
LVEDD			53.0 (4.7)
LVESD			34.1 (3.9)
IVSd			9.5 (1.0)
PWTd			8.9 (0.8)
LV wall motion abnormalities		0	
E/A			1.4 (0.4)
Aortic root			31.0 (2.8)
Ascending aorta			27.4 (3.5)
LAV			37.4 (10.8)
RV wall motion abnormalities		0	
RVD1			36.8 (2.8)
RVD2			29.5 (3.7)
TAPSE			22.9 (2.7)
S’ wave			13.8 (1.2)

LVEDD: Left Ventricular End-Diastolic Diameter; LVESD: Left Ventricular End-Systolic Diameter; IVSd: Interventricular Septum in diastole; PWTd: Posterior Wall Thickness in diastole; LAV: Left Atrial Volume; RVD1: Right Ventricular Basal diameter; RVD2: Right Ventricular Mid diameter; TAPSE: Tricuspid Annular Plane Systolic Excursion.

**Table 2:** Comparison of electrocardiographic features between individuals with and without a diagnosis of cardiovascular disease.

Parameters	No Diagnosis (%)	Diagnosis (%)	*p*-Value
Resting VRA T0	12 (48.0)	20 (71.4)	0.082
Anterior	3 (12.0)	3 (10.7)	1.000
Lateral	6 (24.0)	7 (25.0)	0.933
Inferior	0	1 (3.6)	1.000
Anterior-lateral	0	2 (7.1)	0.492
Inferior-lateral	3 (12.0)	6 (21.4)	0.474
Anterior-Inferior	0	0	-
Global	0	1 (3.6)	1.000
Resting VRA FU	9 (31.0)	20 (71.4)	0.01 *
Anterior	2 (8.0)	2 (7.1)	0.906
Lateral	3 (12.0)	6 (21.4)	0.474
Inferior	0	1 (3.6)	1.000
Anterior-lateral	2 (8.0)	4 (14.3)	0.355
Inferior-lateral	2 (8.0)	4 (14.3)	0.355
Anterior-Inferior	0	0	-
Global	0	3 (10.7)	0.238
Stress VRA T0	25 (100.0)	28 (100.0)	0.148
Anterior	1 (4.0)	1 (3.6)	1.000
Lateral	5 (17.9)	5 (17.9)	1.000
Inferior	2 (7.1)	2 (7.1)	1.000
Anterior-lateral	1 (4.0)	2 (7.1)	0.472
Inferior-lateral	7 (28.0)	8 (28.6)	1.000
Anterior-Inferior	0	1 (3.6)	1.000
Global	9 (32.1)	9 (32.1)	1.000
ST-depression	11 (44.0)	15 (53.6)	0.319
Stress VRA FU	23 (92.0)	27 (96.4)	0.511
Anterior	1 (4.0)	2 (7.1)	0.472
Lateral	1 (4.0)	1 (3.6)	1.000
Inferior	0	0	-
Anterior-lateral	4 (8.0)	1 (3.6)	0.176
Inferior-lateral	16 (64.0)	17 (60.7)	0.658
Anterior-Inferior	0	2 (7.1)	-
Global	1 (4.0)	4 (14.3)	0.426
ST-depression	15 (60.0)	21 (75.0)	0.856
PACs T0	3 (12.0)	2 (7.1)	0.658
PACs FU	6 (24.0)	6 (21.4)	0.823
PVCs T0	4 (16.0)	2 (7.1)	0.404
PVCs FU	7 (28.0)	6 (21.4)	0.521

VRA: Ventricular Repolarization Abnormalities; T0: baseline evaluation; FU: follow-up; PACs: Premature Atrial Contractions; PVCs: Premature Ventricular Contractions. Percentages are within columns unless otherwise specified. * significant values.

## Data Availability

The raw data supporting the conclusions of this article will be made available by the authors on request.
